# Poverty Dynamics and Academic Trajectories of Children of Immigrants

**DOI:** 10.3390/ijerph14091076

**Published:** 2017-09-16

**Authors:** Liwei Zhang, Wen-Jui Han

**Affiliations:** Silver School of Social Work, New York University, 1 Washington Square North, New York, NY 10003, USA; lz1292@nyu.edu

**Keywords:** academic trajectories, children of immigrants, ECLS-K, poverty dynamics

## Abstract

Using Early Childhood Longitudinal Study, Kindergarten Class of 1998–1999 (ECLS-K), we investigated the relationship between poverty and academic trajectories for children in immigrant families in the United States. We used family socioeconomic status (SES) which considers parental education, parental occupation, and family income to define poverty in correspondence with the U.S. federal poverty threshold. Three dimensions of poverty were examined including depth (i.e., not-poor, near-poor, poor or extreme poor), stability (i.e., continuously or intermittently), and duration (i.e., for how many times in poverty). Our results indicated that living in poverty, particularly when it was extreme, volatile, and for long spell could compromise children’s reading and math achievements during the first nine schooling years. Children of immigrants were doing as well as, if not better than, children of native-borns in certain areas (i.e., math) or in facing of certain pattern of poverty (i.e., long-spell). However, deep poverty and volatile changes in family SES could compromise academic achievements for children of immigrants throughout their first nine years of schooling, a period holds important key to their future success. Implications to practice and policy as well as future directions were discussed.

## 1. Introduction

The first years of a child’s life lay the foundation for the development of cognitive skills and academic achievement, critical components of child development. Family investment plays a key role in ensuring optimal child development; however, the ability to do so depends heavily on resources, both within and outside of the family. Since the 1980s, family income inequality in the United States has increased, accompanied by a rising rate of poverty among children [1]. At the same time, we have observed strong inequalities in learning skills at school entry in the U.S. [2,3,4,5]. Indeed, children in the United States experience the largest gaps in school readiness due to family socioeconomic status (SES) compared to children in other developed countries with similar economic scales (e.g., Australia, Canada, and the UK) [6].Numerous studies show that immigrant families with at least one-foreign parent are more likely than their counterparts to face economic hardship (e.g., [7,8]), a serious obstacle to children’s academic success. The U.S. Census projects that by 2040 children with foreign-born parents will comprise about 50% of America's youths, and the majority of these will be Hispanic and Asian [9]. In 2015, nearly one-quarter, or 17.9 million, of all children in the United States had at least one foreign-born parent [10]. Importantly, children of immigrants comprised a substantial share of the school-aged population in 2015, nearly 32%, or 9.47 million children [10]; children of immigrants are defined in this paper to be those who have at least one foreign-born parent and children themselves could be either native-born or foreign-born and immigrant families are defined to be families with at least one foreign-born parent. Longitudinal studies also indicate that children of immigrants are more likely to be exposed to long spell of poverty than their counterparts [11]. Despite the economic challenges, many studies suggest that children of immigrants perform as well as if not better than their peers of native-born families on certain academic skills such as math (e.g., [12,13,14,15]). In the context of poverty and child development, a question remains: How do dimensions of poverty play a role in the academic achievement of children of immigrants? This paper fills this knowledge gap.

In this paper, we used a large, contemporary, and nationally representative cohort of children who entered kindergarten in 1998/99 to examine how poverty dynamics might be associated with the academic trajectories of children of immigrants during their first nine years of schooling. We acknowledge that children’s experience of poverty is diverse and evolves over time. The depth, stability (e.g., changes in SES or poverty status), and duration of spells of poverty can vary greatly. Thus, we take into account the dynamic nature of poverty and the sensitivity of children’s educational outcomes to each of these different dimensions.

### 1.1. Poverty and Academic Achievement

Growing, consistent evidence has shown that cognitive and language development are particularly vulnerable to the impact of poverty during early years of a child’s life [1,16]. The Ecological System Theory posits that human development evolves through dynamic interactions between a changing individual and a changing context [17]. Mounting evidence suggests that the influences of poverty on cognitive development are a function of the accumulation and interaction of risk factors, the individual’s susceptibility to the environment, and the depth, stability, and duration of the exposure to deprivation [1,18].

It is well-documented that poverty is associated with a range of compromised educational outcomes for children in the realms of academic achievement [19,20,21]. Longitudinal studies indicate that children who grow up poor generally start schooling well behind their non-poor peers in terms of academic achievement, and it is challenge for them to catch up with their financially advantaged peers during subsequent school years [21].

Two models help explain how a family’s economic circumstances can affect the children’s academic achievement: the economic investment model and the psychological stress model. The economic investment model emphasizes parents’ material investments in their children’s educational resources [22,23,24]. A substantial body of research suggests that higher family income or SES enables parents to invest more in educational resources and other enrichments (e.g., books, high-quality child care, extracurricular activities), but that families exposed to poverty or to unstable socioeconomic circumstances are less able to do so [25,26,27]. The psychological stress model emphasizes that economic hardships can lead to increased parental stress and thus decrease the quality of parenting and the home environment, which are strong predictors of educational outcomes [28,29].

Although a large body of literature has examined the association between poverty and child academic achievement, the vast majority of such studies conceptualize poverty using a point-in-time measure or averaged income across time due to data at hand. These approaches may mask substantial and distinct patterns of poverty dynamics (e.g., duration and stability) over the course of childhood, which may be associated with different childhood experiences, thereby affecting academic achievement. Recently poverty researchers have started to acknowledge that children may experience poverty differently over time due to changes in the depth, stability, and duration of spells of poverty (e.g., [30,31,32,33]). However, no study has yet examined this issue among children of immigrants.

Looking at various dimensions of poverty allows us to capture a more complete picture of a child’s experience of economic deprivation than focusing on a single point-in-time measure or an average of economic circumstances across several years. Our longitudinal data offer insight into the temporal or systematic dynamics of childhood economic deprivation and related academic outcomes over time. We acknowledge, however, that due to data at hand, we were not able to explore when a child experienced poverty (e.g., early childhood vs. middle-childhood) despite some research suggesting that some developmental stages might be more sensitive to poverty than others [16].

### 1.2. Poverty Dynamics

Since the 2000s, the rate of child poverty in the United States has held steady at 20%, with higher rates among racial minority groups and foreign-borns [8]. Although many children experience stable economic conditions without poverty throughout their childhood, increasing numbers of children in the United States—including those in so-called middle-class families—have experienced volatile family socioeconomic circumstances in recent decades and an increased likelihood of exposure to poverty [31,34,35]. Studies indicate that more than one-third of all children in the United States experience poverty for at least one year during childhood, and nearly 15% of these experience long spells of poverty lasting at least five years [36]. Since 1975, a growing share of the US population is considered to be living in deep poverty, defined as family income below 50% of federal poverty line) [37]. The two primary drivers pushing children into poverty or pulling them out are changes in household earnings (e.g., changes in parental employment status or weekly work hours) and changes in household demographics (e.g., changes in family structure) [21]. Poverty itself presents a risk to child academic achievement, but perhaps more important is the growing volatility in family SES in the United States due to frequent job changes and the rise in irregular work schedules. In the following sections, we discuss three dimensions of poverty that could potentially influence child academic achievement: depth, volatility, and duration.

#### 1.2.1. Depth

One way to conceptualize poverty is to examine the degree to which children are socioeconomically disadvantaged. Some families fall far below the poverty line (e.g., extreme poverty), whereas others live just below (i.e., poor) or just above (i.e., near poor). Due to social safety net requirements, the experiences of the poor and the near-poor can differ substantially despite perhaps only a few dollars difference in family income. Eligibility for many social welfare programs such as Temporary Assistance to Needy Families, housing subsidies, and child care assistance is tied to income. Families with incomes falling just above the poverty line could be more vulnerable than those who are poor if they cannot access social programs, particularly during economic downturns [38]. The experiences of children living in families with incomes just below the poverty line are likely quite different from those living in extreme poverty. Parents’ struggles to provide sufficient food and shelter for children may affect child academic achievement. The depth of the poverty, thus, carries great implications not only for an individual’s daily experiences but also for public policy. To determine appropriate subsidy levels and the types of services needed by children and families, policymakers need detailed data about the depth of family poverty. Studies have shown that simply classifying people as “in poverty” or “not in poverty” is not sufficient [38,39]. The diversity in access to economic resources due to the depth of poverty helps explain the gaps in family investment in children’s education [1].

#### 1.2.2. Volatility

Volatility refers to changes in family income or socioeconomic circumstances, which can stem from changes in parental employment or work hours [34]. Traditionally, the stability of family income or SES has always been an important issue in understanding a family’s experience and thus child academic achievement. Instability typically occurs when a parent loses a job or changes jobs. In recent years, US employers have begun issuing irregular schedules and relying heavily on on-call or stand-by workers, particularly for low-wage and low-skilled jobs [40,41]. Such workers cannot rely on a stable salary, increasing income volatility among families. According to recent studies, almost half of hourly workers have volatile work hours and thus unstable income. For example, a parent who is employed on an hourly pay scale could work anywhere from 15 to 39 h in a week [42,43]. Whereas poverty brings risks to child academic achievement, instability in a family’s economic environment could have an even more profound impact on child academic achievement. A dependable and stable income allows families to consistently invest in their child’s education, such as affording a quality after-school program [31,34]. Fluctuations in family income or SES may also lead to parental stress and anxiety, which can compromise parent-child relationships and parenting quality [16]. In addition, unstable family SES can increase child stress, drawing focus away from school learning [31,44].

Another important factor is the direction of family socioeconomic change. An upward SES change may not have the same impact on child academic achievement as a downward SES change [31]. Studies have shown consistent adverse effects on child academic achievement associated with downward volatile economic circumstances, but results are mixed on the impact of an increase in SES [45]. On the one hand, increased income or SES could benefit children if parents are able to invest more in their children’s learning and schooling. On the other hand, a positive economic change, particularly an irregular one, could disrupt routines (e.g., consistent access to child care, health insurance), bringing chaos to a child’s daily life [16].

#### 1.2.3. Duration

A third dimension of poverty that could affect child academic achievement is the amount of time a child spends living in poverty, often referred to as poverty spells [46]. Studies have consistently found that longer versus shorter spells of poverty are associated with worse academic achievement and reduced educational attainment (e.g., [36,47]). For example, Korenman et al. [47] found that children who experienced a longer versus shorter spell of poverty performed worse on reading and math. Several studies show that persistent economic hardship is associated with chronic lack of access to resources, which may undermine a child’s academic achievement [48,49]. In addition, long-term economic hardship often leads to family and child stress, which again may substantially compromise children’s academic achievement [33].

### 1.3. Poverty and Children of Immigrants

Whereas studies have documented higher likelihood of children of immigrants experiencing socioeconomic deprivation than their counterparts [9,11], recent studies have begun to underscore the poverty experiences between immigrant and native-born families in terms of different dimensions of poverty dynamics. A few studies have found that the newly-arriving immigrant families are more likely to be exposed to deep, unstable household income, and longer-spell of poverty, which may negatively affect child academic achievement (e.g., [11,31,50,51,52]). For example, Latino immigrants, particularly those of Mexican origin, are more likely to experience extreme poverty [51]. Furthermore, families experiencing extreme poverty tend to remain in poverty for longer period of time and are less likely to catch up socioeconomically with their native-born peers despite they may have already been in the United States for long time [51]. In 2014, nearly 27% or 4.8 million children of immigrants had parents without secure employment in the United States, which could put these children at great risk of economic instability [52].

The significant association found between poverty and child academic achievement applies to children of immigrants in general. However, studies also suggest two paradoxical results: on the one hand, poverty may have a stronger adverse effect on academic achievement among children of immigrants than for their counterparts; on the other hand, children of immigrants may do as well as their peers of native-born families in terms of their academic achievements [53]. Two evidence-based perspectives—immigrant risk (e.g., [7]) and the immigrant paradox (e.g., [53])—help explain these mixed findings. The immigrant risk perspective posits that children in immigrant families would be more inhibited academically by the socioeconomic factors [7]. One argument is that immigrant families are more likely to be exposed to multiple forms of risk beyond economic hardship, such as discrimination, limited language fluency, limited access to welfare system or other financial benefits [7,54,55]. The interactions of such risk factors could more strongly affect child academic achievement among immigrants than among other groups [7,52]. For example, studies indicate that children of immigrants may experience discrimination at school both personally and structurally, such as negative interactions with peers or school staff, devaluation of child primary languages by school teachers, misdiagnosis of special educational needs as well as negative views on child school behavior [56], all of which may compromise school learning for children of immigrants. The lack of English fluency could limit child’s communication with peers and teachers, making it even harder for children to access to educational resources [7]. Immigrant families are also more vulnerable to unstable economic circumstances due to limited access to financial services (e.g., range of financial credit, loan options or low-cost financial alternatives) that could help buffer them against economic crises [50].

The immigrant paradox, however, suggests that although children of immigrant are more likely to be exposed to economic hardship compared to their native-born peers, for a variety of reasons they have similar or better educational outcomes [53]. One of the major mechanisms that help cushion children from the impact of poverty on their academic achievement is family value. As suggested in previous studies, the highly selective process of immigration leads to high aspirations for immigrant families to maintain strong supportive family environment and high parental educational expectation for their children, despite facing various socioeconomic challenges [57,58,59,60]. Children of immigrants also share the sense of obligation to their family, with high academic motivations [61]. For example, studies have suggested that children in families with at least one foreign-born parent may be resilient (e.g., strong supportive family environment and higher parental educational expectation), which can help cushion them from the impact of poverty on their academic achievement [62]. Findings from studies using ethnographies or qualitative approaches suggest that children of immigrants may be raised in a family environment that strongly support academic achievements. There is some evidence to show that this is true for children from Central America, Indochina, India, and East Asia [12,13,58,63,64,65,66]. For example, a reading desk for children to properly sit at and do their homework at home is considered to be the most important necessity for Chinese parents, because doing homework on a kitchen or dining table is considered to be a “disrespectful” behavior toward learning. Such behaviors and attitudes demonstrate the importance Chinese parents place in educational achievement as the best way for individuals to experience great personal success and make a significant contribution to society. These serious attitudes and resulting behaviors toward learning held by parents not only translate into high parental expectations and aspirations for their children to achieve the best education they can, but also significantly influences children’s own attitudes and behavior. Evidence from previous studies has identified the great effort and time devoted by immigrant adolescents to doing homework and with the desire to achieve academic success [67].

### 1.4. The Present Study

In sum, the theoretical framework discussed above has offered two opposing hypotheses about the links between poverty and child academic achievements in immigrant families. The literature thus far has not offered clear empirical evidence about whether children of immigrants will do as well as, worse than, or better than their native-born peers in their academic achievement under the circumstances of different depth, stability, and duration of poverty. Both theory and empirical evidence have underscored the importance of using a longitudinal lens with measures of multiple dimensions of poverty to examine the link in immigrant families between children’s experiences of poverty and their academic trajectories. In this paper, we used a large national longitudinal dataset, the Early Childhood Longitudinal Study-Kindergarten (ECLS-K) cohort of 1998/99, to examine the association between different dimensions of poverty (i.e., depth, stability, and duration) and children’s academic trajectories from kindergarten to eighth grade, paying particular attention to children of immigrants. We also considered a rich set of family sociodemographic characteristics, the home environment, and parental educational expectations, all of which are widely considered in the child development fields (e.g., [68]) to be vital to children’s academic achievement.

## 2. Method

### 2.1. Data

The ECLS-K, administered by the U.S. Department of Education’s National Center for Educational Statistics, collected data on a nationally representative cohort of children who entered kindergarten in the fall of 1998 and who were followed longitudinally. As detailed in ECLS-K User’s Manual [69], using a multistage probability design, children were drawn randomly from a nationally representative sample of roughly 1000 U.S. public and private schools, with an average of more than 20 children per school. One hundred primary sampling units (PSUs) were selected, which were geographic areas consisting of counties or groups of counties. The second-stage units within PSUs were schools. Students within sampled schools were the third- and final-stage units. The ECLS–K sample began with 21,260 children in about 800 public and 200 private schools at kindergarten entry in the fall of 1998 and were followed through eighth grade. We used data from all six waves of data collected by the ECLS-K (fall and spring semesters of kindergarten, and spring semester of first, third, fifth, and eighth grade) for data analysis, with a total of approximately 21,260 children included in the study by eighth grade. All reported sample sizes were rounded to the nearest 10 following ECLS-K reporting requirements.

The ECLS–K collected detailed information on each child’s immigration background, family socioeconomic status, and academic achievement at each time point, making it the ideal national dataset for evaluating the relationships among immigrant status, poverty dynamics, and children’s academic trajectories during their first nine school years.

We used direct assessments of children’s reading and math achievement in the spring of kindergarten, spring of first grade, third grade, fifth grade, and eighth grade. Family SES (i.e., household income, parental education, and occupation) was constructed from the parent interview at each time point. We also considered information on family characteristics and parental involvement in home learning and school activities, which was gathered from parents.

Note that 7% of children (mostly those of immigrants) in the ECLS-K did not complete a direct reading assessment because of limited English proficiency at kindergarten year. Due to improving language skills, less than 1% of children did not complete a direct reading assessment by the end of first grade. All students were assessed in English by third grade. In math, all students were assessed in all grades regardless of their language ability.

### 2.2. Participants

Our sample consists of approximately 21,260 children who appeared in the ECLS-K dataset during the sample period. Approximately 22% of the sample were children of immigrants. Among the children of native-borns (i.e., both parents were native-borns), 69% were non-Hispanic White, 16% were non-Hispanic Black, 9% were Hispanic, 2% were Asian, and 5% were from other racial/ethnic groups. About half of the children were males. The sample includes children from diverse socioeconomic backgrounds. The median annual household income was about $40,000 at kindergarten and $50,533 at eighth grade in constant 1998 dollars, ranging from $5000 or less to $200,001 or more annually. Parental highest education ranges from 8th grade or below to doctorate or professional degrees, and parents had more than 22 categories of occupations.

### 2.3. Measures

#### 2.3.1. Academic Achievement

Direct assessments of reading and mathematics competence were collected via one-on-one testing from kindergarten to fifth grade, and in small group settings timed and proctored by trained test administrators at eighth grade, using an item response theory (IRT) approach [69]. Roughly 15% of children identify by teachers or school records as having a non-English language background were administered a brief language screening (the Oral Language Developmental Scale, or OLDS) in the fall of kindergarten to determine their eligibility in taking English version of reading assessment. Approximately 51% or 1010 of these children (7% of the overall sample) scored below the cut-off point and were administered only the mathematics assessments that year. By first grade, this number dropped to 270 (less than 1% of the overall sample). From third grade, all children were assessed in English. Following previous empirical study [70], scores for children who were not administered English version of the reading assessment were imputed with multiple imputation as detailed below. The assessment included a few items that almost all children would get wrong and a few that almost all children would get right in each assessment, so that floor and ceiling effects were avoided [71]. To assure that data was comparable over time, the same assessment instruments were used in kindergarten and first grade, with several new items included for third grade and fifth grade, and again for eighth grade.

Standardized t scores (M = 50, SD = 10) were computed to examine reading and math scores via a transformed measure of the IRT scale score. The standardized t score was a norm-referenced score that could represent children’s abilities relative to their average peers nationwide (i.e., children who entered kindergarten in fall 1998), and a change in mean t scores over time reflects a change in relative ability [71], which is the focus of this paper.

- *Reading (language and literacy).* Basic reading skills such as letter and word recognition and receptive vocabulary were measured at the kindergarten and first grade. The third- and fifth-grade reading assessments included items designed to measure grade-appropriate comprehension such as phonemic awareness, vocabulary, passage comprehension, and some of the more difficult test items from the earlier assessments. The eighth-grade reading assessment focused on measuring skills such as forming a general understanding of the text, developing a more complete understanding of what was read, and critically evaluating, comparing and contrasting, and understanding the effect of literary devices or the author’s intentions [71].
- *Math.* Skills in conceptual and procedural knowledge and problem solving were measured in kindergarten and first grade, with about half of the math assessment including questions on number sense, number properties, and operations. The other half of the assessment included questions on such as measurement, geometry and spatial sense, statistics and probability, and patterns [71]. The third-, fifth- and eighth-grade math assessments measured the same content areas as first grade but with a larger emphasis on problem solving.

#### 2.3.2. Immigrant Status

Immigrant status was determined by the parent’s answer to the question of whether he or she was born in the United States and the country from which he or she came. The questions were asked in the first, third, and fifth grade to both parents. Children were coded as “children of immigrant” if they had at least one foreign-born parent, and coded as “children of native-born” if both parents were born in the United States.

#### 2.3.3. Poverty Dynamics

We used both the SES measure and household income to define family poverty status. Because the results were similar for SES and income measures, we present SES results in the paper for brevity. In addition, the measure of SES more fully captures the family’s socioeconomic status than just focusing on income. The SES variable was calculated from household income, parental education, and parental occupation at each time point from kindergarten to eighth grade, with a mean of 0 and standard deviation of 1. Three dimensions of poverty dynamics were examined including depth (i.e., not-poor, near-poor, poor or extreme poor), stability (i.e., the direction of changes in SES and poverty status), and duration (i.e., number of times in poverty since kindergarten). Previous research has used the two-year average percentage change in income/SES to assess the stability of poverty (e.g., [72,73]). It was calculated as 100 × (Y_t_ − Y_t−1_)/Y_average_ with Y_average_ = (Y_t_ + Y_t−1_)/2 [73]. The two-year average percent change has two advantages over simple percent change. It has symmetric values in terms of increases (positive value) and decreases (negative value). And it is naturally bounded between 200% and −200% [73]. We did not find any significant effects associated with this measure in our analyzed sample; thus, we do not present results using this measure, but these analyses are available upon request.

*Depth.* We divided our sample into four poverty groups—not-poor, near-poor, poor, and extremely poor—based on the sample distribution of SES for each wave respectively (e.g., above −0.03, above −0.38 but at or below −0.03, above −0.74 but at or below −0.38, at or below −0.74, at kindergarten). These four cut-off points at each wave were consistent with the U.S. federal poverty threshold in defining people into different income poverty groups: household income above 200% of the federal poverty threshold (“not-poor”), above 100% but at or below 200% of the federal poverty threshold (“near-poor”), above 50% but at or below 100% of the federal poverty line (“poor”), and at or below 50% of the federal poverty line (“extremely poor”). For example, respondents with SES ranging from −0.38 to −0.03 at kindergarten wave who were identified as “near-poor” also had their household income put them into “near-poor” category based on the U.S. federal poverty threshold.

- *Stability.* We used two measures to examine poverty stability: direction of SES change and direction of poverty status change. First, we determined whether a child’s family had experienced increased, stable, or decreased SES between two time points. The SES change was defined as a decrease if the two-year average SES percentage change was at or below −33% and as an increase if it was at or above 33%. If the absolute value of the two-year average percentage change was less than 33%, we classified the direction of SES change as stable. The threshold of 33% was chosen following prior research [72]. We also performed growth curve analysis using various thresholds for SES change, including 25%, 33%, 45% and 50%, as sensitivity check. Similar results were obtained for the different thresholds. We also classified the direction of poverty status change into three categories: improved, stable, or worsened. To determine this variable, we examined whether the household experienced a positive or negative transition between the four types of poverty status: not-poor, near-poor, poor, and extremely poor. The direction of poverty status change was coded as improved (worsened) if there was a positive (negative) change in family poverty status between two consecutive waves. If the child’s poverty status stayed the same, this variable was coded as stable. For instance, if a child’s poverty status changed from poor to near-poor or not-poor, the direction of poverty status change would be classified as improved.
- *Duration.* We examined poverty duration by counting the times children were exposed to poverty (i.e., poor or extreme poor). This is a discrete variable ranging from zero to five across the five time points from kindergarten to eighth grade. We used times rather than years to examine the number of spells during which children were exposed to poverty because the data were not collected every year and the exact intervals between two waves could vary between children.

#### 2.3.4. Child and Family Characteristics

Both time-invariant and time-variant variables were included to capture child and family characteristics. A set of time-invariant variables from the fall of kindergarten included child’s gender, birth weight, race/ethnicity (i.e., non-Hispanic White, non-Hispanic Black, Hispanic, Asian, and other), mother’s marital status at birth, and attendance at pre-K center-based care. Time-variant variables at all interview points included the living in a single-parent family, the number of people under age 18 in the household, presence of siblings, parental educational expectations (ranged from 1 = less than high school to 6 = PhD, MD, or other higher degree), home learning activities (e.g., a scale indicating the frequency of reading books to the child at home, telling stories, visiting museums, Cronbach alpha = 0.60), parental school involvement (e.g., volunteering at school, attending PTA meetings, and participating in school fundraising; Cronbach alpha = 0.60), and location (city, suburban, or rural) and region (e.g., Northeast) of residence.

### 2.4. Empirical Strategy

We used three-level growth-curve modelling to examine the associations between immigrant status, poverty dynamics, and the child’s academic trajectories, with longitudinal data at six assessment points for reading and math. Data were analyzed with Level 1 as time (i.e., within-individual effects), Level 2 as individuals (i.e., between-individual and within-school effects), and Level 3 as schools (i.e., between-school effects). Growth-curve models with three-levels are able to allow the researchers to compare the rate of change of each group (i.e., which have faster or slower change rates over time). Three-level growth-curve models also partition the outcome variance into between- and within-school portions, allowing for more accurate standard error estimates to account for students being nested within schools [74].

Attrition rates ranged from 10% (at spring kindergarten wave) to 55% (at eighth grade) with sample sizes started with about 21,260 at kindergarten to about 9720 at eighth grade, which was expected for a longitudinal study [69]. For the time-invariant sociodemographic variables, we used the data collected at kindergarten, with rates of missing data generally less than 8% (e.g., low birth weight). The missing rate for parents’ country of birth, which was collected at first, third, and fifth grade, were generally less than 14% at the three time points. For the time-varying sociodemographic variables, the missing rates ranged from 1 to 15% (e.g., parental educational expectations) at kindergarten and from 3 to 10% (e.g., home learning environment) at eighth grade. For children’s reading and math scores, the missing rates were 11% and 8%, respectively, at kindergarten, and about 5% for both at eighth grade. Caution thus is needed when interpreting the results due to missing data particularly on reading assessment at kindergarten and first grade on some children. Pattern of missing data were examined using Pearson correlation [75,76] and the results indicate some significant associations between the missing-data indicator (i.e., reading score missing) and other explanatory variables (e.g., the largest correlation coefficients were r = 0.39, *p* < 0.001, for the association between the missingness of reading score at kindergarten and primary home language is not English; all other correlation coefficients were smaller than 0.20). Missing at random (MAR) assumes that the missingness of the data is related to the observed data and is independent of the unobserved data (i.e., missing data itself). Based on the ECLS-K data collection process [69], the missingness of the reading scores at kindergarten and first grade depends on child’s non-English background, rather than child’s actual reading skills [75,76]. In other words, children who did not take the reading test did not necessarily have worse reading scores. The assumption that the data were missing at random is thus not rejected. Though attrition and missing cases are unavoidable, the growth-curve modelling used in this analysis handles unbalanced data well, because students did not have to be assessed at all data points to be included in the analysis [77]. Still, we used multiple imputation (with STATA’s “ice” command, taking multi-level data into consideration) to handle missing data (including outcome variables) with five imputed data sets [75]. The command “mi estimate: mixed” was then used to conduct the growth curve analysis.

We followed the recommendation in the longitudinal data analysis literature to conduct a sequence of statistical models [77]. We first examined the unconditional means model to assess the amount of outcome variation that exists at each level. Next we examined the unconditional growth model to assess the extent to which within-person variation is systematically associated with time. Predictors were then added with immigrant status and the interaction between immigrant status and time (so that the growth rates of the two groups were allowed to differ). The next models further added the predictors of each dimension of poverty dynamics (i.e., depth, direction of SES change, direction of poverty status, and duration) in separate models and the interactions with time. The next models further added controls for child and family characteristics for each outcome as the final model. These models allowed us to examine the variation in child academic achievement as a function of immigrant status and poverty dynamics net of other important factors. To further explore how the relationship between poverty dynamics and children’s academic achievement were differentiated by immigrant status, we conducted interaction analyses between each dimension of poverty dynamics and children’s immigrant status. Specifically, we interacted three categorical poverty variables—depth, direction of SES change, and direction of poverty status change—with immigrant status to create mutually exclusive dichotomous groups for each of the three interaction models. For the continuous poverty variable, duration, we added the product term of poverty duration with immigrant status to examine the interaction effects. The final models were conducted separately to examine the main and interaction effects on each child academic outcome.

The Akaike Information Criterion (AIC) and the Bayesian Information Criterion (BIC) indicated that the last two sets of models—the main effect models and interaction effect models with controls of family and child characteristics—provided the best fit for the data. For brevity, we present only the final models. All continuous variables of child and family characteristics were centered at their grand mean values except the dummy variables (e.g., gender) so that the reference child represents a realistic scenario [77]. In addition, the time variable was centered according to the true starting point in each case, so that the initial status refers to kindergarten for reading and math. To avoid the potential problem of multicollinearity, all of the analyses were conducted separately for each dimension of poverty dynamics. In all analyses with categorical poverty variables (i.e., depth, direction of SES change and direction of poverty status), the reference group was children who were socioeconomically advantaged (i.e., not poor, SES stable, poverty status stable). In the analyses with continuous poverty variables (i.e., duration), the value of zero refers to never having been exposed to poverty during the sample period.

We first present a descriptive picture of the sample characteristics by their immigrant status. We then show our growth curve results on the associations between immigrant status, poverty dynamics, and academic trajectories by controlling for sociodemographic characteristics. Finally, we present growth curve results on the association between poverty dynamics and academic trajectories moderated by immigrant status.

## 3. Results

### 3.1. Descriptive Picture

Table 1 presents sample characteristics by immigrant status. For brevity, we show only the descriptives at eighth grade for time-varying variables. About 22% of the sample were children of immigrants. In general, compared to children of native-borns, children of immigrants tended to be more financially disadvantaged but had similar, if not better, sociodemographic backgrounds than their counterparts. Specifically, children of immigrants were more likely to be exposed to poverty, have unstable SES, and have experienced a long-spell of poverty compared to children of native-borns. Children of immigrants were less likely to have attended center-based care before entering kindergarten and less likely to reside in single-parent families. They were more likely to have mothers married at birth, have siblings, and have more persons under age 18 in the household. For parental educational practices and home environment, immigrant parents were more likely to have higher educational expectations for their children, but they were less likely to participate in school events. In addition, children with immigrant parents tended to have fewer home learning activities compared to their counterparts. In addition, children of immigrants tended to live in the West in urban or suburban areas, whereas their native-born peers tended to live in the Midwest or the South and in rural areas. Finally, the raw data indicate that children of immigrants performed worse in their reading and math at eighth grade than their native-born counterparts.

Table 2 displays in more detail the distribution of each dimension of poverty dynamics by immigrant status at each time point from kindergarten to eighth grade. In general, compared to children of native-borns, children of immigrants were more likely to be poor, to have more volatile family SES, and to have experienced a long spell of poverty during their first nine years of schooling. About 20 to 25% of the children of immigrants saw their family SES increase between kindergarten and eighth grade, but the degree of increase may not have been large enough to bring them out of poverty once and for all, as about 45% of all children of immigrants experienced more than one spell of poverty throughout the first nine years of their schooling.

Table 3 presents children’s academic achievement from kindergarten to eighth grade by both immigrant status and dimension of poverty dynamics. In general, compared to their peers, children who were socioeconomically disadvantaged (exposed to poverty, unstable socioeconomic circumstances, and chronic poverty) tended to perform worse on their reading and math scores from kindergarten to eighth grade. Specifically, for both immigrants and native-borns, children who were not poor tended to have the highest scores on reading and math compared to children from all levels of poverty, and children living in extreme poverty (family income below 50% of the federal poverty line) had the lowest scores on both reading and math. Children with volatile family SES tended to have lower academic scores than their counterparts, regardless of whether family SES increased or decreased. The picture was the same for children whose poverty status improved or worsened. The longer children stayed in poverty, the lower their academic achievement, and children who experienced poverty four or more times from kindergarten to eighth grade had the lowest academic achievement.

### 3.2. Growth-Curve Analysis

The descriptive statistics confirm that living in poverty—particularly when it is extreme, volatile, and for a long spell—may compromise children’s academic achievement during the first nine years of schooling. We next assess children’s academic trajectories from kindergarten to eighth grade. For brevity, we present only the final model specification that includes all sociodemographic characteristics described in Measures section along with immigrant status and poverty dynamics. Table 4 presents the academic achievement results for both reading and math. Four models are presented for each outcome, corresponding to each of the four dimensions of poverty dynamics.

#### 3.2.1. Reading

Results for children’s reading trajectories from kindergarten to eighth grade from both the unconditional means model and the unconditional growth model (not shown, available upon request) indicate that the average child’s reading score was 49.32 (*p* < 0.001) and had a negative slope of −0.12 (*p* < 0.01). Thus, reading scores decreased through the grades, on average. 

In addition, the average child’s reading scores varied significantly over time, with approximately 42% of the variation in reading scores attributable to differences among children and 27% attributable to differences among schools.

Results on reading shown in Table 4 (left columns) expand on the unconditional growth model by adding immigrant status and four dimensions of poverty dynamics as predictors, as well as sociodemographic characteristics as controls. These models provide controlled answers to (1) whether immigrant status makes difference in children’s reading scores and (2) how each dimension of poverty dynamics shapes children’s reading achievements differently over time.

Across all four models, the coefficient estimates for immigrant status indicate that children of immigrants had worse reading scores than children of native-borns at kindergarten entry, and the gap between the two groups became larger over time (i.e., statistically significantly negative slope), once we control for poverty dynamics and sociodemographic backgrounds. For example, in Model 1, children of immigrants were 1.33 points lower than their native-born peers at kindergarten entry; by the end of eighth grade, the former group lagged farther behind with 1.85 (−1.33 + (−0.13 × 4)) points.

The four models on reading in Table 4 also examine the associations between each dimension of poverty dynamics and initial status and rates of change in reading scores, while controlling for immigrant status and sociodemographic characteristics. Results indicate that children who were socioeconomically disadvantaged had worse reading scores at kindergarten entry and lagged farther behind children who were not socioeconomically disadvantaged over time. Results in Model 1 indicate that deeper poverty was significantly associated with lower reading score and decreasing rates of change over time. For example, the average reading score for children who were not poor at kindergarten entry was significantly higher than children who were near poor (*b* = −1.09, *p* < 0.001), poor (*b* = −1.50, *p* < 0.001), and extremely poor (*b* = −1.56, *p* < 0.001). In addition, the poorer the children were, the larger the differentials in rates of change between the not-poor and the near-poor (*b* = −0.34, *p* < 0.001), the poor (*b* = −0.75, *p* < 0.001), and the extremely poor (*b* = −1.13, *p* < 0.001). In Model 2 of reading in Table 4, the coefficient estimates indicate that although unstable family SES was significantly associated with higher reading score at kindergarten entry (*b* = 0.61, *p* < 0.001), it had significant decreasing rates of change over time (*b* = −0.38, *p* < 0.001) compared to children whose family SES was stable. Results in Model 3 of Table 4 indicate that children whose poverty status changed over time had similar reading scores at kindergarten entry as children whose poverty status remained stable, but they had significantly decreasing rates of change on their reading scores, regardless of whether their poverty status improved (*b* = −0.32, *p* < 0.001) or worsened (*b* = −0.36, *p* < 0.01). Results in Model 4 suggest that long-spell poverty was significantly associated with worse reading scores (*b* = −0.93, *p* < 0.001) and decreasing rates of change (*b* = −0.16, *p* < 0.001).

Within-person variance remained similar across models; this was expected as only a few time-varying Level 1 predictors were added. In addition, the variance components were lower than the unconditional growth model. Overall, the final models explained 4–5% of the variation in between-person initial status, 1–2% of the variation in between-person rates of change, 21–25% of the variation in between-school initial status, and 1–7% of the variation in between-school rates of change. To evaluate the fit of models in regard to reading, we used three goodness-of-fit indices [77]: the deviance statistic, Akaike’s information criterion (AIC), and the Bayesian information criterion (BIC). For each model, the decrease in the deviance statistic was significant at *p* < 0.001, indicating that the final model considering all variables provided the best fit compared with the unconditional growth model (not shown, available upon request).

#### 3.2.2. Math

The unconditional-means and unconditional-growth model results (not shown, available upon request) for math indicate that the average child’s math score was 49.59 (*p* < 0.001) with a strong negative slope of −0.14 (*p* < 0.001), indicating that math scores decreased from kindergarten to eighth grade. In addition, the average child’s math score varied significantly over time, with about 45% of the variation in math scores attributable to differences among children and 27% to differences among schools.

The four models on math in Table 4 are the results of expansions to the unconditional-growth model from adding immigrant status, each dimension of poverty dynamics as predictors, and child and family characteristics as controls. These models provide controlled answers to: (1) whether children’s math scores vary as a function of immigrant status and (2) how each dimension of poverty dynamics shapes children’s math scores over time.

The coefficient estimates of immigrant status across the four models indicate that children of immigrants had significantly lower math scores at kindergarten entry, compared to children with native-born parents. However, they had significantly faster rates of change, narrowing the initial gap with their native-born peers by eighth grade. For example, in Model 1, after controlling for poverty depth and sociodemographic variables, the initial difference in math between the two groups was −2.65 (*p* < 0.001) at kindergarten entry, but that difference dropped to −0.53 (−2.65 + (0.53 × 4)) by eighth grade.

The four models also present the controlled estimate of the associations between poverty dynamics and math trajectories, suggesting that children who were socioeconomically disadvantaged had worse math scores at kindergarten entry than those who were not socioeconomically disadvantaged. The gaps in math scores became larger between the poverty groups over time, controlling for immigrant status and sociodemographic background. Specifically, in Model 1, the initial math differences were −1.00 (*p* < 0.001), −1.56 (*p* < 0.001), and −1.93 (*p* < 0.001), respectively, for near-poor, poor, and extremely poor, compared to children who were not poor. By eighth grade, the differences had grown to −2.20 (−1.00 + (−0.30 × 4)), −3.32 (−1.24 + (−0.52 × 4)), and −4.57 (−1.93 + (−0.66 × 4)), respectively, suggesting that the poorer the children were, the lower their math scores were and the faster their rates of decrease were over time. Results in Models 2 and 3 indicate that compared to children who had stable family SES or stable poverty status, decreasing family SES was significantly associated with higher math scores at kindergarten entry, but unstable family SES and volatile poverty status (no matter increasing or decreasing) were both significantly associated with faster rates of decrease in math scores. Results in Model 4 suggest that long-spell poverty was significantly associated with lower math scores (*b* = −1.02, *p* < 0.001) and decreasing rates of change (*b* = −0.10, *p* < 0.001).

Much as with the reading models, there was no reduction in within-person variance across models due to the lack of time-varying Level 1 predictors. The variance components in each model were lower than the unconditional growth model. Immigrant status, poverty dynamics, and child and family characteristics in the final model explained 4–5% of the variation in between-person initial status, 2–3% of the variation in between-person rates of change, 29–34% of the variation in between-school initial status, and 3–5% of the variation in between-school rates of change. The three goodness-of-fit indices presented at the bottom of Table 4 (the deviance statistic, AIC, and BIC) indicate that the final model, which controls for all variables, provided a better fit than the growth-means model (not shown, available upon request).

#### 3.2.3. Interaction between Immigrant Status and Poverty Status

Results in Table 4 indicate that both immigrant status and poverty dynamics compromised children’s academic achievement to various degrees. Deep, volatile, and long duration poverty were particularly associated with lower, if not the lowest, academic achievement. However, children of immigrants did not necessarily do worse, at least in math. Below, we explore children’s academic achievement by considering both immigrant and poverty dynamics. In all regression specifications, children of native-borns who were not poor, who had stable SES or poverty status, or who did not experience any poverty were the reference group.

- *Reading.* The left columns of Table 5 expand on Table 4 by examining how being immigrant and being poor might be associated with children’s reading trajectories, controlling for child and family characteristics. Results indicate that children of immigrants who were socioeconomically disadvantaged performed significantly worse in reading compared to children of native-borns who were not socioeconomically disadvantaged but also compared to children of native-borns with similar socioeconomic disadvantages. The poorer these children were, the more unstable their family’s socioeconomic condition, and the longer they stayed in poverty, the worse their reading scores were at kindergarten entry compared to their corresponding SES groups of native-borns. However, children of immigrants had similar decreasing rates of changes over time as their peers with native-born parents with similar socioeconomically disadvantaged conditions; consequently, children of immigrants did not lag farther behind over time. In addition, children of immigrants had significantly increasing rates of change (i.e., improving reading scores) the longer they stayed in poverty over time compared to their peers of native-borns.
  Specifically, in Model 1 of Table 5 (left columns), the coefficient estimates show that the average reading score for non-poor children of native-borns was 49.57 (*p* < 0.001) at kindergarten entry, with significantly increasing rates of change (indicating improving reading scores) over time (*b* = 0.20, *p* < 0.001). Children of immigrants who were poor, regardless of degree of poverty (near poor, poor, and extreme poor), had the worst reading scores—significantly lower than both the non-poor and corresponding poor children of native-borns. For example, among children who were in ‘poor’ group, children of native-borns had an average reading score of 48.46 (49.57 − 1.11) at kindergarten entry and 46.06 ((49.57 − 1.11) + (0.20 − 0.80) × 4) by eighth grade, whereas for children of immigrants, the corresponding figures were 46.07 (49.57 − 3.50) and 43.75 ((49.57 − 3.50) + (0.20 − 0.78) × 4). The difference in coefficients between poor children of native-borns and immigrants was statistically significant at kindergarten entry (i.e., −1.11 vs. −3.50, *p* < 0.001), with similar rates of change over time (i.e., −0.80 vs. −0.78, ns). These results apply to the comparisons between children of native-borns and immigrants within the near-poor or extremely poor groups. Results in Model 2 suggest that compared to children of native-borns, the association between unstable SES change and reading score was more pronounced for children of immigrants. Specifically, among children who experienced a large SES decrease, children of native-borns had significantly higher reading scores than children of immigrants at kindergarten entry (i.e., 0.68 vs. −1.07, *p* < 0.001). For example, among children whose family experienced a large SES decrease, the average reading score for a child of native-born parents at kindergarten was 48.88 (48.20 + 0.68), and the score for a child of immigrants was 47.13 (48.20 − 1.07). These two groups of children had similar rates of change over time (i.e., −0.39 vs. −0.66, ns). By eighth grade, the figures were 47.16 ((48.20 + 0.68) + (−0.04 − 0.39) × 4) for the former and 44.33 ((48.20 − 1.07) + (−0.04 − 0.66) × 4) for the latter. Results in Model 3 suggest stronger associations between the direction of poverty status change and reading scores for children of immigrants than for those with native-born parents. For example, among those whose poverty status worsened, the average reading score for children of native-borns decreased from 48.84 (48.29 + 0.55) at kindergarten entry to 46.68 ((48.29 + 0.55) + (0.02 − 0.56) × 4) by eighth grade, whereas the figures were 46.74 (48.29 − 1.55) and 44.62 ((48.29 − 1.55) + (0.02 − 0.55) × 4) for children of immigrants. The gaps between the two groups were significant at kindergarten entry (i.e., 0.55 vs. −1.55, *p* < 0.001), with similar rates of change over time (i.e., −0.56 vs. −0.55, ns). Results in Model 4 reveal a paradoxical pattern: Although both children of immigrants and children of native-borns who experienced long spells of poverty had significantly lower reading scores at kindergarten entry than children of native-borns who did not experience any poverty, the former group had significantly increasing rates of change (*b* = 0.16, *p* < 0.001) from kindergarten to eighth grade, whereas the latter had significantly decreasing rates of change (*b* = −0.20, *p* < 0.001). For example, for children of immigrants who experienced five spells of poverty, their reading scores changed from 42.65 (49.85 − 0.15 + (−0.71 × 5) + (−0.70 × 5)) at kindergarten entry to 41.21 (49.85 − 0.15 + (−0.71 × 5) + (−0.70 × 5) + (0.17 − 0.33 +(−0.20 × 5) + (0.16 × 5)) × 4) by eighth grade, whereas the reading scores for children of native-borns who experienced five spells of poverty were 46.30 (49.85 + (−0.71 × 5)) at kindergarten entry, dropping to 42.98 (49.85 + (−0.71 × 5) + (0.17 +(−0.20 × 5)) × 4) by eighth grade.
- *Math.* The right columns of Table 5 present the interaction analysis on math scores. Overall, the results suggest that the associations between poverty and children’s math scores were more pronounced for children of immigrants at kindergarten entry. However, children of immigrants who were socioeconomically disadvantaged closed the gaps with children with native-born parents who were not socioeconomically disadvantaged, while children of native-borns who were socioeconomically disadvantaged lagged farther behind children with native-born parents who were not socioeconomically disadvantaged. In Model 1, poor children of immigrants at all depths of poverty had significantly lower math scores at kindergarten entry than not only non-poor children of native-borns but also poor children of native-borns at all depths of poverty. However, poor children of immigrants closed the gaps with non-poor children of native-borns by eighth grade. In contrast, poor children of native-borns lagged farther behind their non-poor peers with native-born parents. For example, near-poor children of immigrants had significantly lower math scores at kindergarten entry than their near-poor peers with native-born parents (i.e., −3.67 vs. −0.86, *p* < 0.001). However, the former group had significantly different (or faster) rates of change over time than the latter (i.e., 0.16 vs. −0.31, *p* < 0.001). The average math score for near-poor children of native-borns decreased from 48.16 (49.02 − 0.86) at kindergarten entry to 46.60 ((49.02 − 0.86) + (−0.08 − 0.31) × 4) by eighth grade, whereas the figures for near-poor children of immigrants were respectively 45.35 (49.02 − 3.67) and 45.67 ((49.02 − 3.67) + (−0.08 + 0.16) × 4). We found similar results when comparing children of native-borns and immigrants who were poor or extremely poor. Results in Model 2 suggests that among children who experienced the same negative SES change, children of immigrants had significantly worse math scores at kindergarten entry (i.e., −2.68 vs. 0.62, *p* < 0.001) but significantly increasing rates of change (i.e., 0.18 vs. −0.30, *p* < 0.001) in math than their native-born counterparts. Specifically, the coefficient estimates in Model 2 indicate that among children who experienced a large downward SES shock, math scores for children of native-borns decreased from 48.30 (47.68 + 0.62) at kindergarten entry to 46.10 ((47.68 + 0.62) + (−0.25 − 0.30) × 4) by eighth grade, whereas the corresponding figures for children of immigrants were 45.00 (47.68 − 2.68) and 44.72 ((47.68 − 2.68) + (−0.25 + 0.18) × 4). Similar comparison results apply to groups of children whose SES increased. Similarly, in Model 3, among children whose poverty status worsened, children of immigrants had significantly worse math scores at kindergarten entry (i.e., −3.48 vs. 0.63, *p* < 0.001)) but significantly increasing rates of change (i.e., 0.58 vs. −0.50, *p* < 0.001) compared to their native-born counterparts. Results in Model 4 indicate that children of immigrants who experienced a more spells of poverty had significantly lower math scores at kindergarten entry but a significantly increasing rate of change compared to children of native-borns experiencing a similar spells of poverty. For example, the average math score at kindergarten entry of a child of immigrants who experienced five spells of poverty was 41.24 (49.45 − 1.76 + (−0.89 × 5) + (−0.40 × 5)), increasing to 41.88 (49.45 − 1.76 + (−0.89 × 5) + (−0.40 × 5) + (−0.11 + 0.42 + (−0.13 × 5) + (0.10 × 5)) × 4) by eighth grade; but for a child of native-borns who similarly experienced five times of poverty, the figures were 45.00 (49.45 + (−0.89 × 5)) at kindergarten entry and 41.96 (49.45 + (−0.89 × 5) + (−0.11 + (−0.13 × 5)) × 4) by eighth grade. These results show that the academic achievement gaps in math closed significantly over time.

## 4. Discussion and Conclusions

Many previous studies have looked at poverty as a monolithic state: either a child is or is not living in poverty. Given the extensive literature showing the connection between poverty and child academic achievement, we aimed to provide a more nuanced assessment of how poverty dynamics may be associated with the academic trajectories of children of immigrants from kindergarten to eighth grade. Thus, in our analyses, we included measures of the depth, stability, and duration of spells of poverty that children experienced during their first nine years of schooling.

Our bottom-line findings are consistent with those of the previous studies: that poverty has adverse impacts on children’s academic achievement (e.g., [19,21]). Our results indicate that living in poverty, particularly when it is extreme, volatile, or for a long spell could compromise children’s reading and math performance both at kindergarten entry and through the eighth grade. Children who were socioeconomically disadvantaged not only had worse reading and math scores at kindergarten entry, but they also lagged farther behind children who were not socioeconomically disadvantaged over time. These findings applied to both children of native-borns and children of immigrants.

Importantly, our findings shed light on the complexities of poverty dynamics in relation to academic trajectories for children of immigrants. Informed by two opposing hypotheses—immigrant risk and the immigrant paradox, our findings somewhat support both hypotheses. Although children of immigrants performed worse at kindergarten entry than their peers with native-born parents in the face of extreme, volatile, and long-spell poverty, they did not always lag farther behind during the first nine years of schooling. In many cases, children of immigrants had significantly faster paces of learning, particularly in math, closing the gap with their corresponding peers with native-born parents by eighth grade.

We find some support for the immigrant-risk hypothesis. Specifically, children of immigrants in each poverty group had significantly lower reading and math scores at kindergarten entry than both non-poor and poor peers with native-born parents. Children of immigrants were particularly vulnerable to deep and unstable socioeconomic circumstances, which supports prior research documenting the limited socioeconomic resources (e.g., financial access, family asset) available to immigrant families [50,78,79]. Socioeconomic resources help shield children and families from the disruptions brought on by extreme poverty or economic crises. In addition, children of immigrants may also face other risks beyond economic hardships, such as language barriers and discrimination. It is thus not surprising that children of immigrants living in poverty faced more challenges in school, particularly in reading, which depends heavily on language skills. Our raw data also showed that children of immigrants were not only more likely to be exposed to deep, volatile, and long spells of poverty, but also less likely to get access to some critical socioeconomic resources both before school entry (e.g., less center-based care before entering kindergarten) and during the nine years of schooling (e.g., lower quality of home environment), which serve as buffers against the adverse impacts of poverty.

We also find some evidence supporting the immigrant paradox hypothesis. Despite facing poverty (whether near-poor, poor, or extremely poor), large downward changes in SES and poverty status, and long spells of poverty, children of immigrants in our sample over time were able to either not fall farther behind or at times close the gaps in academic achievement with their peers with native-born parents, particularly in math. This finding is consistent with prior studies showing that children of immigrants perform as well as, if not better than, their native-born peers on math (e.g., [12,13,14,15]). For example, Han [70] found that in spite of generally disadvantaged family backgrounds, children with non-English language backgrounds had increasing growth rates in math scores, which may suggest the important role of resources other than family SES in shaping these children’s academic trajectories (e.g., school). Although beyond the scope of this paper, children of immigrants might be more resilient to adverse circumstances (e.g., SES violate and long-spell poverty) than their native-born peers. Such resilience may have to do with their family environment such as higher parental educational expectations that help offset the adverse effects associated with poverty [60].

Our analysis built upon and extended the current literature on the importance of examining how poverty dynamics might be associated with the academic trajectories of children of immigrants. Consistent with prior research [16], the depth, stability, and duration of poverty, when examined together, revealed a consistent picture of how the academic achievement of the children of immigrants can suffer during the first nine years of schooling. Our results highlight the complex associations between family socioeconomic fluctuations and children’s academic outcomes. Previous studies have found that downward volatile economic circumstances are associated with adverse effects on child academic achievement, but the results are mixed for how upward volatile economic change can affect children [45]. Our findings add to this literature, indicating that even upward socioeconomic change could have adverse effects on children’s academic achievement. Our raw data suggest that families who experienced improved SES/poverty status between two time periods also tended to be those who had relatively less advantaged backgrounds (e.g., child having low birth weight, living in single-parent families, lower parental educational expectations, and lower quality of home environment), which might explain the decreasing rates of academic performance. It is also likely that despite these families experienced upward SES/poverty status change, the improvement might not large enough (e.g., from being “extreme poor” to “poor” or from “poor” to “near poor”) to bring them out of poverty and into secure socioeconomic status. In addition, our findings may also speak to the importance of stability and consistency for children’s optimal academic achievement during the early school years. Many educational or family routines, such as regular access to afterschool programs or health insurance, rely on consistent family investment. Temporary upward socioeconomic changes may lead to more harm than good [16].

### Limitations

Despite the longitudinal nature of our dataset, this study does have some limitations. First, we adopted several measures to tap different dimensions of poverty, including depth, stability, and duration, but these do not adequately and accurately capture children’s poverty experiences. We built upon and expanded the current literature on poverty dynamics, but acknowledge that more nuanced approaches are needed. For example, due to data at hand, we could only examine SES or poverty status at each time point of data collection instead of monthly or even annually. Family socioeconomic circumstances can change seasonally, monthly, or even weekly, and a more detailed dataset could refine our understanding of children’s experiences [72]. Future research will benefit from more detailed data on children’s socioeconomic backgrounds.

Second, the present study may be biased due to missing information in the dataset. The sample started with about 21,260 children in kindergarten, decreasing to about 9720 children in eighth grade due to attrition [69]. Even though such a decrease is expected for a longitudinal dataset and the data were imputed, it would nevertheless have been preferable to have actual assessment data. In addition, some children, primarily children of immigrants, did not complete the reading assessment in kindergarten and first grade due to limited English proficiency. If these children had scored poorly or better on the reading exam, the analyses might have underestimated the disadvantages or advantages for children of immigrants.

Third, although this study considered a rich set of covariates, it is challenging to partial out the effects of poverty or family SES from related socioeconomic disadvantages to which children might be exposed. As suggested in previous studies, poverty or low SES are largely linked to less-than-perfect family environments (e.g., poor parental mental well-being) as well as school-level resources (e.g., school quality, percentage of students receiving free lunch), which are also predictive of children’s academic achievement [70,80]. Future research is warranted to take both the family-level and school-level factors into account in order to gain a fuller picture of the links between poverty dynamics and child academic achievements. Relatedly, while this study examined the associations between poverty dynamics and academic trajectories of children of immigrants, we were not able to assess the mechanisms behind these associations. For example, our data did not include information about why a family was living in poverty, which could affect how the family and children cope with the economic crisis [16]. Also, given that deep, volatile, and long-spell poverty compromised children’s academic trajectories, it is expected that addressing these facets of poverty would likely lead to improved children’s academic achievement. Our understanding would benefit from examining pathways (e.g., school investment, parenting, public policies) through which different patterns of poverty might compromise or benefit academic achievement. It would also be important to pay attention to the factors and pathways that are important to children of immigrants. The immigrant-risk and immigrant-paradox hypotheses suggest that immigrant families experience obstacles possess resilience that differ from those of native-born families. Our study highlights the importance of contextual factors in shaping individual children’s learning, in this case, immigrant status. Future research could extend our study by focusing on other contextual factors such as race/ethnicity, country of origin, and culture that might help shape children’s learning experiences in the face of different patterns of poverty.

Despite these limitations, this study offers valuable empirical evidence on the complexities of different dimensions of poverty in shaping children’s academic trajectories throughout their first nine years of schooling, particularly for children of immigrants. The overall findings from this study indicate that poverty, particularly deep, volatile, and long-spell poverty, was associated with lower, if not the lowest, academic performance for children. We also found that children of immigrants were doing as well as, if not better than, children with native-born parents in certain areas (i.e., math) and in the face of particular patterns of poverty (i.e., long-spell poverty). However, deep poverty and volatile socioeconomic changes were shown to compromise academic achievement during the first nine years of schooling, a period that sets the stage for future success. These findings speak to the importance of considering various patterns of poverty when devising policies and programs that address the strengths of and challenges facing children of immigrants. This study is a first step in addressing children’s dynamic poverty experiences, particularly those of children of immigrants. We acknowledge that poverty is a complex experience that could have different meanings for each child and family, and a child’s response to poverty can vary greatly. Further studies are needed to explore the still under-researched issues such as how to accurately measure different patterns of poverty over time, and how those patterns of poverty may shape children’s academic achievement in various sociodemographic contexts or through different pathways.

## Figures and Tables

**Table 1:** Child and family characteristics by immigrant status (*N* ≈ 21,410).

Analyzed Variables	Total	Non Immigrant	Immigrant	Sig.
Immigrant (%)	22.04	0.00	100.00	***
Race/Ethnicity of Native-born (%)				
Non-Hispanic White	53.91	69.15	0.00	***
Non-Hispanic Black	12.24	15.70	0.00	***
Hispanic	6.79	8.71	0.00	***
Asian	1.23	1.58	0.00	***
Others	3.79	4.87	0.00	***
*Poverty Dynamics At/By Eighth Grade*				
Depth (%)				
Not-Poor	56.78	60.98	42.06	***
Near-Poor	20.94	21.88	17.63	***
Poor	13.83	11.86	20.77	***
Extreme Poor	8.44	5.28	19.54	***
Direction of SES Change (%)				
Stable	65.61	66.38	62.87	**
Increased	18.27	17.80	19.96	*
Decreased	16.12	15.82	17.17	ns
Direction of Poverty Status Change (%)				
Stable	82.90	83.52	80.68	**
Improved	10.15	9.71	11.72	*
Worsened	6.95	6.77	7.60	ns
Duration (%)				
0 time in poverty	60.94	64.90	46.94	***
1 time in poverty	9.07	9.44	7.76	**
2 times in poverty	9.97	9.66	11.08	*
3 times in poverty	7.91	7.17	10.51	***
4 times in poverty	5.70	4.43	10.18	***
5 times in poverty	6.40	4.39	13.53	***
Total Times in Poverty from Kindergarten to 8th Grade	1.08 (1.60)	0.90 (1.46)	1.70 (1.90)	***
*Child and Family Characteristics*				
Time-Invariant (at Kindergarten)				
Boy (%)	51.16	51.21	50.99	ns
Low birth weight (<2500 g) (%)	7.09	7.07	7.19	ns
Center-based care before entering kindergarten (%)	83.45	84.11	80.30	***
Mother married at birth (%)	73.53	72.75	76.34	***
Time-Varying (at Eighth Grade)				
Residing in single-parent family (%)	20.33	21.52	16.14	***
Having siblings (%)	84.60	83.37	88.89	***
Number of persons age <18 in the household	2.33 (1.12)	2.29 (1.09)	2.49 (1.20)	***
Parental Educational Practices and Home Environment				
Educational expectation	4.09 (1.07)	3.99 (1.01)	4.45 (1.19)	***
Participating in school events	0.00 (0.60)	0.01 (0.59)	−0.05 (0.62)	***
Home learning activities	0.00 (0.45)	0.03 (0.43)	−0.10 (0.48)	***
Region of Residence (%)				
Northeast	18.44	18.68	17.61	ns
Midwest	27.95	32.14	13.37	***
South	32.73	34.05	28.11	***
West	20.88	15.12	40.91	***
Local (%)				
City	33.22	28.75	49.21	***
Suburban	40.31	40.04	41.28	***
Rural	26.47	31.21	9.51	***
*Academic Achievement at Eighth Grade*				
Reading	50.02 (9.99)	50.49 (9.78)	48.40 (10.55)	***
Math	50.01 (9.99)	50.23 (9.80)	49.28 (10.61)	***

*Note.* Chi-square was used for categorical variables and *t*-test was used for continuous variables for the comparison between children in immigrant and non-immigrant families. * *p* < 0.05, ** *p* < 0.01, *** *p* < 0.001.

**Table 2:** Sample size and percentage distribution of poverty DYNAMICS by immigrant status from kindergarten to eighth grade.

Poverty Status Over Time	Non-Immigrant (*n* ≈ 12,290)	Immigrant (*n* ≈ 3270)	Total (*n* ≈ 15,560)
*Depth*			
Kindergarten			
Not-Poor ***	51.07%	39.27%	48.57%
Near-Poor ***	21.08%	13.97%	19.57%
Poor	18.49%	19.43%	18.69%
Extreme Poor ***	9.37%	27.33%	13.17%
1st Grade			
Not-Poor ***	52.46%	38.76%	49.59%
Near-Poor ***	23.48%	16.63%	22.04%
Poor ***	15.79%	20.33%	16.75%
Extreme Poor ***	8.27%	24.28%	11.62%
3rd Grade			
Not-Poor ***	55.60%	40.15%	52.19%
Near-Poor ***	22.31%	17.08%	21.15%
Poor ***	16.02%	24.18%	17.82%
Extreme Poor ***	6.07%	18.59%	8.84%
5th Grade			
Not-Poor ***	56.61%	38.70%	52.26%
Near-Poor ***	23.27%	19.30%	22.30%
Poor ***	13.36%	21.66%	15.37%
Extreme Poor ***	6.76%	20.34%	10.06%
8th Grade			
Not-Poor ***	60.98%	42.06%	56.78%
Near-Poor ***	21.88%	17.63%	20.94%
Poor ***	11.86%	20.77%	13.83%
Extreme Poor ***	5.28%	19.54%	8.44%
Direction of SES Change			
Kindergarten to 1st Grade			
Stable	54.26%	55.97%	54.61%
Increased ***	27.03%	22.76%	26.16%
Decreased ***	18.70%	21.27%	19.23%
1st Grade to 3rd Grade			
Stable	78.29%	78.63%	78.36%
Increased *	3.36%	4.24%	3.55%
Decreased	18.35%	17.12%	18.09%
3rd Grade to 5th Grade			
Stable *	70.71%	68.05%	70.10%
Increased ***	14.97%	18.44%	15.78%
Decreased	14.32%	13.51%	14.13%
5th Grade to 8th Grade			
Stable **	66.38%	62.87%	65.61%
Increased *	17.80%	19.96%	18.27%
Decreased	15.82%	17.17%	16.12%
Direction of Poverty Status Change			
Kindergarten to 1st Grade			
Stable ***	74.84%	77.75%	75.44%
Improved *	14.91%	13.21%	14.56%
Worsened *	10.25%	9.05%	10.01%
1st Grade to 3rd Grade			
Stable *	78.02%	75.98%	77.59%
Improved ***	12.22%	15.83%	12.99%
Worsened *	9.76%	8.19%	9.43%
3rd Grade to 5th Grade			
Stable **	84.54%	81.99%	83.95%
Improved *	8.57%	10.25%	8.96%
Worsened	6.89%	7.76%	7.09%
5th Grade to 8th Grade			
Stable **	83.52%	80.68%	82.90%
Improved *	9.71%	11.72%	10.15%
Worsened	6.77%	7.60%	6.95%
*Duration*			
Kindergarten			
0 time in poverty ***	72.14%	53.24%	68.14%
1 time in poverty ***	27.86%	46.76%	31.86%
K to 1st Grade			
0 time in poverty ***	68.57%	50.17%	64.60%
1 time in poverty	12.25%	13.43%	12.51%
2 times in poverty ***	19.17%	36.40%	22.89%
K to 3rd Grade			
0 time in poverty ***	66.40%	48.21%	62.44%
1 time in poverty	10.47%	9.55%	10.27%
2 times in poverty ***	11.65%	16.26%	12.65%
3 times in poverty ***	11.48%	25.98%	14.64%
K to 5th Grade			
0 time in poverty ***	65.52%	47.26%	61.50%
1 time in poverty	9.50%	8.52%	9.28%
2 times in poverty ***	10.29%	12.09%	10.69%
3 times in poverty ***	7.91%	12.71%	8.97%
4 times in poverty ***	6.78%	19.41%	9.56%
K to 8th Grade			
0 time in poverty ***	64.90%	46.94%	60.94%
1 time in poverty **	9.44%	7.76%	9.07%
2 times in poverty *	9.66%	11.08%	9.97%
3 times in poverty ***	7.17%	10.51%	7.91%
4 times in poverty ***	4.43%	10.18%	5.70%
5 times in poverty ***	4.39%	13.53%	6.40%

*Note.* Poverty status was adjusted with family SES based on family income and federal poverty threshold. Not-Poor: Household income >200% of federal poverty threshold; Near-Poor: Household income >100% & ≤200% of federal poverty threshold; Poor: Household income >50% & ≤100% of federal poverty threshold; Extreme Poor: Household income ≤50% of federal poverty threshold. Chi-square was used for the comparison between children in immigrant and non-immigrant families. * *p* < 0.05, ** *p* < 0.01, *** *p* < 0.001.

**Table 3:** Child academic achievement from kindergarten to eighth grade by immigrant status and poverty dynamics.

Immigrant and Poverty Status	Reading							Math						
	Kindergarten	First Grade	Third Grade	Fifth Grade	Eighth Grade	Score Change	% Change	Kindergarten	First Grade	Third Grade	Fifth Grade	Eighth Grade	Score Change	% Change
Total	50.26 (9.99)	50.20 (10.02)	50.19 (9.97)	50.09 (10.00)	50.02 (9.99)	−0.24	−0.48	50.38 (10.03)	50.20 (10.02)	50.15 (10.00)	50.06 (10.00)	50.01 (9.99)	−0.37	−0.73
*Depth*														
Non Immigrant														
Not-Poor	52.87 (10.21)	53.66 (9.89)	54.70 (8.67)	54.47 (8.32)	53.62 (7.97)	0.75	1.42	54.60 (10.14)	54.42 (9.79)	54.40 (8.91)	54.06 (8.18)	53.45 (7.95)	−1.15	−2.11
Near-Poor	49.29 (8.25)	49.4 (8.78)	49.96 (9.07)	49.83 (8.93)	48.76 (9.29)	−0.53	−1.08	50.01 (8.72)	49.81 (9.09)	49.73 (9.09)	49.39 (9.13)	48.60 (9.43)	−1.41	−2.82
Poor	46.74 (7.10)	46.68 (8.07)	46.31 (9.70)	45.96 (10.02)	44.73 (10.47)	−2.01	−4.30	47.06 (8.11)	46.96 (9.09)	45.82 (9.13)	45.54 (10.09)	44.43 (10.49)	−2.63	−5.59
Extreme Poor	44.88 (7.37)	43.48 (8.19)	41.84 (9.71)	40.29 (10.61)	39.76 (10.99)	−5.12	−11.41	43.99 (7.10)	42.93 (7.81)	41.25 (8.29)	39.34 (10.3)	39.39 (10.61)	−4.60	−10.46
Immigrant														
Not-Poor	54.86 (13.14)	54.61 (10.98)	54.37 (8.65)	54.25 (8.27)	54.30 (7.61)	−0.56	−1.02	53.97 (10.81)	53.48 (10.24)	54.36 (9.54)	54.52 (8.76)	54.34 (8.41)	0.37	0.69
Near-Poor	48.68 (7.72)	48.25 (8.40)	47.29 (8.79)	47.16 (9.24)	48.06 (9.53)	−0.62	−1.27	47.64 (8.38)	47.43 (7.87)	48.33 (8.73)	49.10 (9.17)	48.87 (9.83)	1.23	2.58
Poor	46.34 (7.55)	45.46 (7.64)	44.52 (8.79)	44.79 (9.30)	44.25 (10.60)	−2.09	−4.51	44.27 (7.81)	45.46 (8.31)	46.05 (8.77)	46.24 (10.03)	45.54 (10.64)	1.27	2.87
Extreme Poor	44.85 (5.55)	43.63 (6.90)	41.01 (8.06)	41.29 (8.84)	40.85 (10.15)	−4.00	−8.92	42.85 (7.19)	44.08 (7.72)	43.54 (8.69)	43.83 (9.65)	42.99 (10.99)	0.14	0.33
*Stability—Direction of SES Change*														
Non Immigrant														
Stable	51.40 (9.92)	51.89 (9.90)	52.76 (9.47)	52.45 (9.29)	51.69 (9.12)	0.29	0.56	52.79 (10.00)	52.54 (10.09)	52.39 (9.58)	52.01 (9.28)	51.38 (9.27)	−1.41	−2.67
Increased	50.07 (9.39)	50.35 (9.84)	50.62 (9.93)	50.32 (10.15)	49.27 (10.14)	−0.80	−1.60	50.84 (9.66)	50.71 (9.63)	50.29 (9.86)	49.74 (10.16)	49.32 (9.97)	−1.52	−2.99
Decreased	50.78 (9.33)	51.14 (9.83)	51.37 (9.96)	50.88 (10.06)	50.20 (10.12)	−0.58	−1.14	51.80 (10.39)	51.54 (10.26)	51.16 (9.93)	50.58 (10.08)	50.01 (10.12)	−1.79	−3.46
Immigrant														
Stable	51.33 (11.54)	50.39 (10.55)	49.11 (10.24)	48.99 (10.19)	49.13 (10.49)	−2.20	−4.29	49.15 (10.58)	49.45 (9.91)	49.80 (10.18)	50.18 (10.21)	49.82 (10.73)	0.67	1.36
Increased	50.14 (10.44)	48.67 (10.01)	47.43 (9.88)	47.88 (9.99)	47.67 (10.62)	−2.47	−4.93	48.23 (9.94)	48.25 (9.73)	48.75 (9.76)	49.01 (10.09)	49.21 (10.35)	0.98	2.03
Decreased	50.62 (11.93)	49.70 (10.19)	47.67 (10.01)	47.94 (10.47)	47.74 (10.75)	−2.88	−5.69	48.36 (10.32)	48.21 (10.04)	49.41 (10.27)	49.15 (10.53)	48.16 (10.87)	−0.20	−0.41
*Stability—Direction of Poverty Status Change*														
Non Immigrant														
Stable	51.58 (9.93)	52.11 (9.95)	52.91 (9.46)	52.58 (9.36)	51.86 (9.15)	0.28	0.54	52.96 (10.13)	52.72 (10.11)	52.54 (9.63)	52.10 (9.39)	51.55 (9.29)	−1.41	−2.66
Improved	48.02 (8.40)	48.06 (9.21)	47.97 (9.63)	47.76 (10.06)	46.33 (10.07)	−1.69	−3.52	48.52 (8.73)	48.49 (9.08)	48.09 (9.4)	47.64 (9.95)	46.75 (10.13)	−1.77	−3.65
Worsened	48.99 (8.03)	48.79 (8.59)	48.79 (10.01)	48.28 (9.86)	47.21 (10.41)	−1.78	−3.63	49.37 (8.74)	48.89 (9.00)	48.24 (9.30)	47.85 (9.85)	47.12 (9.90)	−2.25	−4.56
Immigrant														
Stable	51.74 (11.96)	50.68 (10.74)	49.25 (10.35)	49.18 (10.31)	49.13 (10.63)	−2.61	−5.04	49.48 (10.70)	49.77 (10.07)	50.14 (10.26)	50.34 (10.20)	49.94 (10.70)	0.46	0.93
Improved	47.97 (8.56)	46.57 (8.55)	45.62 (9.10)	45.95 (9.74)	46.11 (10.32)	−1.86	−3.88	45.89 (8.95)	45.60 (9.25)	46.86 (9.60)	47.09 (10.52)	46.81 (10.80)	0.92	2.00
Worsened	46.74 (5.43)	47.05 (7.35)	45.46 (8.07)	46.35 (8.87)	46.72 (9.66)	−0.02	−0.04	46.22 (7.96)	46.02 (7.20)	47.14 (8.25)	47.85 (9.55)	47.69 (9.72)	1.47	3.18
*Duration*														
Non Immigrant														
0 times in poverty	52.11 (10.16)	52.56 (9.97)	53.53 (8.90)	53.51 (8.41)	53.03 (8.21)	0.92	1.77	53.19 (10.01)	53.04 (9.84)	53.16 (9.17)	53.01 (8.49)	52.82 (8.2)	−0.37	−0.70
1 times in poverty	48.13 (8.06)	48.22 (8.86)	48.06 (9.22)	47.84 (9.66)	47.86 (9.6)	−0.27	−0.56	48.63 (8.46)	48.19 (8.86)	47.84 (9.32)	47.79 (9.61)	47.41 (9.55)	−1.22	−2.51
2 times in poverty	46.47 (7.29)	46.13 (8.57)	46.12 (9.37)	46.70 (9.78)	46.01 (10.23)	−0.46	−0.99	46.23 (7.83)	46.07 (8.66)	45.64 (9.33)	45.79 (10.00)	45.90 (9.94)	−0.33	−0.71
3 times in poverty	46.01 (7.16)	45.42 (8.18)	44.53 (9.25)	44.37 (10.14)	44.22 (10.67)	−1.79	−3.89	45.76 (7.86)	45.63 (8.56)	44.71 (8.85)	44.61 (9.95)	44.35 (10.75)	−1.41	−3.08
4 times in poverty	45.49 (6.49)	45.29 (7.87)	44.57 (9.30)	43.76 (9.97)	42.92 (10.6)	−2.57	−5.65	45.39 (7.24)	45.17 (8.11)	44.00 (8.82)	43.22 (10.13)	42.69 (10.59)	−2.70	−5.95
5 times in poverty	45.81 (7.46)	44.88 (8.36)	43.96 (10.00)	43.42 (10.88)	42.41 (10.8)	−3.40	−7.42	45.47 (8.18)	45.05 (9.21)	43.67 (9.17)	42.66 (10.84)	41.90 (10.99)	−3.57	−7.85
Immigrant														
0 times in poverty	53.90 (12.68)	53.57 (10.59)	52.88 (9.13)	52.93 (8.85)	53.61 (7.94)	−0.29	−0.54	52.74 (10.7)	52.22 (10.11)	52.95 (9.91)	53.36 (9.20)	53.72 (8.56)	0.98	1.86
1 times in poverty	47.52 (7.48)	46.50 (8.17)	45.04 (9.24)	45.45 (9.63)	46.66 (9.81)	−0.86	−1.81	46.44 (7.70)	46.62 (8.58)	47.03 (9.52)	47.55 (9.41)	47.84 (9.69)	1.40	3.01
2 times in poverty	46.78 (7.63)	46.58 (8.21)	45.30 (8.75)	46.27 (9.12)	46.46 (10.01)	−0.32	−0.68	45.11 (8.36)	45.14 (8.38)	46.54 (9.56)	48.13 (10.04)	47.79 (10.70)	2.68	5.94
3 times in poverty	45.10 (6.71)	44.37 (7.40)	42.95 (8.34)	43.71 (8.67)	44.36 (9.67)	−0.74	−1.64	44.16 (7.81)	44.25 (7.59)	44.43 (8.44)	45.44 (9.41)	46.39 (9.43)	2.23	5.05
4 times in poverty	46.01 (6.64)	44.74 (8.01)	43.04 (8.78)	42.87 (9.63)	43.29 (10.56)	−2.72	−5.91	44.29 (8.35)	44.79 (8.36)	44.96 (9.34)	44.97 (10.53)	44.63 (10.90)	0.34	0.77
5 times in poverty	45.74 (7.23)	44.63 (7.39)	42.79 (8.86)	43.05 (9.36)	42.40 (10.65)	−3.34	−7.30	43.37 (7.71)	45.00 (8.28)	44.81 (8.88)	45.14 (9.77)	44.06 (11.04)	0.69	1.59

*Note.* Poverty status was adjusted with family SES based on family income and federal poverty threshold. Not-Poor: Household income >200% of federal poverty threshold; Near-Poor: Household income >100% & ≤200% of federal poverty threshold; Poor: Household income >50% & ≤100% of federal poverty threshold; Extreme Poor: Household income ≤50% of federal poverty threshold. Poverty dynamics are shown for by or at eighth grade.

**Table 4:** Growth-curve results of academic achievements from kindergarten to eighth grade.

Variables	Reading				Math			
	Model 1	Model 2	Model 3	Model 4	Model 1	Model 2	Model 3	Model 4
**Fixed effects**								
Intercept	49.67 (0.33) ***	48.21 (0.29) ***	48.29 (0.29) ***	49.97 (0.29) ***	49.09 (0.29) ***	47.69 (0.28) ***	47.76 (0.27) ***	49.52 (0.27) ***
Immigrant	−1.33 (0.19) ***	−1.54 (0.19) ***	−1.51 (0.19) ***	−0.93 (0.19) ***	−2.65 (0.19) ***	−2.94 (0.19) ***	−2.92 (0.19) ***	−2.21 (0.19) ***
*(1) Depth (Reference group: Not-Poor)*								
Near-Poor	−1.09 (0.14) ***				−1.00 (0.12) ***			
Poor	−1.50 (0.16) ***				−1.56 (0.13) ***			
Extreme Poor	−1.56 (0.18) ***				−1.93 (0.16) ***			
*(2) SES Direction (Reference group: SES Stable)*								
SES Increased		0.06 (0.19)				0.07 (0.27)		
SES Decreased		0.61 (0.17) ***				0.52 (0.22) *		
*(3) Poverty Direction (Reference group: Stable Poverty Status)*								
Improved Poverty Status			0.02 (0.18)				0.07 (0.19)	
Worsened Poverty Status			0.35 (0.20)				0.28 (0.23)	
*(4) Duration*								
Times in poverty				−0.93 (0.05) ***				−1.02 (0.05) ***
**Rate of Change**								
Intercept	0.18 (0.05) ***	−0.03 (0.04)	0.00 (0.04)	0.13 (0.04) **	−0.10 (0.04) *	−0.25 (0.04) ***	−0.21 (0.04) ***	−0.13 (0.04) ***
Immigrant	−0.13 (0.07) *	−0.29 (0.07) ***	−0.29 (0.06) ***	−0.14 (0.06) *	0.53 (0.06) ***	0.43 (0.06) ***	0.44 (0.06) ***	0.54 (0.06) ***
*(1) Depth (Reference group: Not-Poor)*								
Near-Poor	−0.34 (0.06) ***				−0.30 (0.06) ***			
Poor	−0.75 (0.07) ***				−0.52 (0.07) ***			
Extreme Poor	−1.13 (0.09) ***				−0.66 (0.10) ***			
*(2) SES Direction (Reference group: SES Stable)*								
SES Increased		−0.13 (0.10)				−0.06 (0.12)		
SES Decreased		−0.38 (0.09) ***				−0.29 (0.12) *		
*(3) Poverty Direction (Reference group: Stable Poverty Status)*								
Improved Poverty Status			−0.32 (0.08) ***				−0.28 (0.09) **	
Worsened Poverty Status			−0.36 (0.10) **				−0.22 (0.11)	
*(4) Duration*								
Times in poverty				−0.16 (0.02) ***				−0.10 (0.01) ***
**Child and Family Characteristics**								
Race/Ethnicity of Native-born (Reference Group: Non-Hispanic White)								
Non-Hispanic Black	−3.29 (0.17) ***	−3.46 (0.18) ***	−3.42 (0.18) ***	−3.29 (0.17) ***	−4.69 (0.18) ***	−4.89 (0.18) ***	−4.85 (0.18) ***	−4.68 (0.18) ***
Asian	0.33 (0.37)	0.17 (0.37)	0.23 (0.36)	−0.07 (0.35)	0.18 (0.34)	−0.03 (0.33)	0.02 (0.33)	−0.19 (0.33)
Hispanic	−1.69 (0.18) ***	−1.84 (0.19) ***	−1.79 (0.19) ***	−1.89 (0.19) ***	−2.50 (0.19) ***	−2.70 (0.19) ***	−2.66 (0.19) ***	−2.70 (0.18) ***
Others	−1.76 (0.27) ***	−1.77 (0.27) ***	−1.78 (0.27) ***	−1.78 (0.27) ***	−2.38 (0.28) ***	−2.46 (0.29) ***	−2.45 (0.29) ***	−2.38 (0.28) ***
Boy	−1.63 (0.09) ***	−1.60 (0.09) ***	−1.60 (0.09) ***	−1.63 (0.09) ***	1.10 (0.09) ***	1.11 (0.09) ***	1.12 (0.09) ***	1.10 (0.09) ***
Low birth weight (<2500 g)	−1.00 (0.21) ***	−1.02 (0.22) ***	−1.02 (0.22) ***	−0.99 (0.20) ***	−1.73 (0.23) ***	−1.75 (0.24) ***	−1.75 (0.24) ***	−1.72 (0.23) ***
Center-based care before entering kindergarten	1.40 (0.15) ***	1.73 (0.16) ***	1.71 (0.16) ***	1.28 (0.16) ***	1.42 (0.14) ***	1.71 (0.14) ***	1.70 (0.14) ***	1.26 (0.14) ***
Mother married at birth	1.96 (0.13) ***	2.46 (0.12) ***	2.42 (0.12) ***	1.77 (0.12) ***	2.08 (0.13) ***	2.55 (0.13) ***	2.51 (0.13) ***	1.84 (0.14) ***
Residing in single-parent family	−0.34 (0.09) ***	−0.65 (0.09) ***	−0.64 (0.09) ***	−0.46 (0.09) ***	−0.20 (0.09) *	−0.48 (0.09) ***	−0.48 (0.09) ***	−0.30 (0.09) **
Having siblings	0.17 (0.12)	0.23 (0.13)	0.20 (0.12)	0.24 (0.12)	0.38 (0.12) **	0.42 (0.13) **	0.41 (0.13) **	0.43 (0.13) **
Number of persons age <18 in the household	−0.57 (0.04) ***	−0.63 (0.04) ***	−0.62 (0.04) ***	−0.55 (0.04) ***	−0.26 (0.04) ***	−0.31 (0.05) ***	−0.31 (0.04) ***	−0.23 (0.04) ***
Parental Educational Practices and Home Environment								
Educational expectation	0.73 (0.03) ***	0.80 (0.03) ***	0.80 (0.03) ***	0.71 (0.03) ***	0.63 (0.03) ***	0.69 (0.03) ***	0.69 (0.03) ***	0.61 (0.03) ***
Participating in school events	0.46 (0.07) ***	0.59 (0.07) ***	0.58 (0.07) ***	0.44 (0.07) ***	0.47 (0.06) ***	0.58 (0.07) ***	0.58 (0.06) ***	0.45 (0.07) ***
Home learning activities	0.57 (0.07) ***	0.77 (0.07) ***	0.77 (0.07) ***	0.59 (0.07) ***	0.59 (0.07) ***	0.75 (0.07) ***	0.75 (0.07) ***	0.58 (0.07) ***
Region of Residence (Reference Group: South)								
Northeast	−0.14 (0.21)	−0.01 (0.23)	−0.01 (0.23)	−0.26 (0.21)	−0.81 (0.21) ***	−0.68 (0.22) **	−0.68 (0.21) **	−0.95 (0.20) ***
Midwest	0.01 (0.19)	0.10 (0.21)	0.09 (0.21)	−0.11 (0.19)	−0.02 (0.18)	0.11 (0.19)	0.10 (0.19)	−0.14 (0.18)
West	−0.70 (0.21) **	−0.63 (0.23) **	−0.64 (0.23) **	−0.70 (0.21) **	−0.90 (0.20) ***	−0.84 (0.21) ***	−0.85 (0.21) ***	−0.92 (0.20) ***
Local (Reference Group: City)								
Suburban	0.68 (0.17) ***	0.94 (0.18) ***	0.93 (0.18) ***	0.61 (0.17) ***	0.70 (0.15) ***	0.92 (0.16) ***	0.91 (0.16) ***	0.60 (0.15) ***
Rural	−0.39 (0.21)	−0.48 (0.22) *	−0.48 (0.22) *	−0.26 (0.20)	−0.71 (0.21) **	−0.80 (0.22) ***	−0.80 (0.22) ***	−0.56 (0.21) **
**Variance Components**								
Within-person	4.50 (0.02) ***	4.47 (0.02) ***	4.48 (0.02) ***	4.46 (0.02) ***	4.32 (0.02) ***	4.29 (0.02) ***	4.29 (0.02) ***	4.28 (0.02) ***
*Level 2-between person*								
In initial status	7.86 (0.05) ***	7.94 (0.06) ***	7.93 (0.06) ***	7.89 (0.05) ***	7.57 (0.05) ***	7.66 (0.05) ***	7.66 (0.05) ***	7.60 (0.05) ***
In rate of change	2.08 (0.03) ***	2.09 (0.03) ***	2.09 (0.03) ***	2.08 (0.03) ***	1.48 (0.03) ***	1.48 (0.03) ***	1.48 (0.03) ***	1.47 (0.03) ***
Covariance	−0.52 (0.01) ***	−0.50 (0.01) ***	−0.51 (0.01) ***	−0.51 (0.01) ***	−0.36 (0.01) ***	−0.34 (0.01) ***	−0.34 (0.01) ***	−0.35 (0.01) ***
*Level 3-between school*								
In initial status	3.49 (0.11) ***	3.59 (0.11) ***	3.59 (0.11) ***	3.40 (0.11) ***	3.25 (0.10) ***	3.37 (0.10) ***	3.38 (0.10) ***	3.15 (0.10) ***
In rate of change	1.08 (0.04) *	1.14 (0.04) ***	1.13 (0.04) ***	1.08 (0.04) *	1.01 (0.03)	1.02 (0.03)	1.02 (0.03)	1.00 (0.03)
Covariance	−0.64 (0.03) ***	−0.52 (0.03) ***	−0.53 (0.03) ***	−0.65 (0.03) ***	−0.61 (0.03) ***	−0.52 (0.03) ***	−0.53 (0.03) ***	−0.62 (0.03) ***
**Model Fit Statistics**								
Deviance (=−2 log-likelihood)	512,725.0	495,917.5	513,863.2	512,394.8	506,718.5	490,256.3	507,614.6	506,258.3
AIC	512,797.0	495,985.5	513,931.2	512,458.8	506,790.5	490,324.3	507,682.6	506,322.3
BIC	513,129.1	496,297.8	514,244.8	512,754.0	507,122.6	490,636.7	507,996.2	506,617.5
R-Squared	0.1909	0.1732	0.1768	0.1971	0.1962	0.1781	0.1810	0.2010

*Note.* Standard errors are in parentheses. Model also controls for child’s characteristics: being a boy, race/ethnicity (i.e., non-Hispanic Black, Hispanic, Asian and other racial/ethnic group), low birth weight, and attending center-based care before kindergarten; family characteristics: mother married at birth, having siblings present, number of family members under age 18 at home, living in single-parent family, parental educational expectations, parental participating in school events, home learning activities, and region and location of residence. AIC = Akaike’s information criterion; BIC = Bayesian information criterion. * *p* < 0.05, ** *p* < 0.01, *** *p* < 0.001.

**Table 5:** Academic achievements from kindergarten to eighth grade by poverty dynamics and immigrant status.

Immigrant Status by Poverty Status	Reading				Math			
	Model 1	Model 2	Model 3	Model 4	Model 1	Model 2	Model 3	Model 4
**Fixed effects**								
Intercept	49.57 (0.33) ***	48.20 (0.29) ***	48.29 (0.29) ***	49.85 (0.29) ***	49.02 (0.29) ***	47.68 (0.28) ***	47.75 (0.27) ***	49.45 (0.27) ***
*(1) Interaction Between Immigrant & Depth (Reference Group: Non Immigrant & Not-Poor)*								
Non Immigrant & Near-Poor	−0.88 (0.14) ***				−0.86 (0.13) ***			
Non Immigrant & Poor	−1.11 (0.18) ***				−1.32 (0.15) ***			
Non Immigrant & Extreme Poor	−0.98 (0.20) ***				−1.50 (0.21) ***			
Immigrant & Not-Poor	−0.26 (0.24)				−1.92 (0.24) ***			
Immigrant & Near-Poor	−2.42 (0.32) ***				−3.67 (0.29) ***			
Immigrant & Poor	−3.50 (0.32) ***				−4.59 (0.27) ***			
Immigrant & Extreme Poor	−3.71 (0.33) ***				−5.19 (0.30) ***			
*(2) Interaction Between Immigrant & SES Direction (Reference group: Non Immigrant & SES Stable)*								
Non Immigrant & SES Increased		0.10 (0.21)				0.19 (0.27)		
Non Immigrant & SES Decreased		0.68 (0.17) ***				0.62 (0.21) **		
Immigrant & SES Stable		−1.52 (0.20) ***				−2.89 (0.19) ***		
Immigrant & SES Increased		−1.64 (0.38) ***				−3.27 (0.47) ***		
Immigrant & SES Decreased		−1.07 (0.35) **				−2.68 (0.40) ***		
*(3) Interaction Between Immigrant & Poverty Direction (Reference group: Non Immigrant & Stable Poverty Status)*								
Non Immigrant & Improved Poverty Status			0.22 (0.21)				0.29 (0.19)	
Non Immigrant & Worsened Poverty Status			0.55 (0.24) *				0.63 (0.27) *	
Immigrant & Stable Poverty Status			−1.47 (0.19) ***				−2.86 (0.19) ***	
Immigrant & Improved Poverty Status			−1.72 (0.45) ***				−2.95 (0.36) ***	
Immigrant & Worsened Poverty Status			−1.55 (0.44) ***				−3.48 (0.36) ***	
*(4) Interaction Between Immigrant & Duration*								
Immigrant				−0.15 (0.21)				−1.76 (0.21) ***
Times in Poverty				−0.71 (0.06) ***				−0.89 (0.05) ***
Immigrant * Times in Poverty				−0.70 (0.10) ***				−0.40 (0.09) ***
**Rate of Change**								
Intercept	0.20 (0.05) ***	−0.04 (0.04)	0.02 (0.04)	0.17 (0.04) ***	−0.08 (0.04)	−0.25 (0.04) ***	−0.19 (0.04) ***	−0.11 (0.04) **
*(1) Interaction Between Immigrant & Depth (Reference Group: Non Immigrant & Not-Poor)*								
Non Immigrant & Near-Poor	−0.36 (0.06) ***				−0.31 (0.06) ***			
Non Immigrant & Poor	−0.80 (0.08) ***				−0.57 (0.08) ***			
Non Immigrant & Extreme Poor	−1.20 (0.11) ***				−0.74 (0.11) ***			
Immigrant & Not-Poor	−0.30 (0.09) **				0.41 (0.08) ***			
Immigrant & Near-Poor	−0.54 (0.12) ***				0.16 (0.11)			
Immigrant & Poor	−0.78 (0.11) ***				0.10 (0.11)			
Immigrant & Extreme Poor	−1.15 (0.11) ***				−0.02 (0.10)			
*(2) Interaction Between Immigrant & SES Direction (Reference group: Non Immigrant & SES Stable)*								
Non Immigrant & SES Increased		−0.15 (0.10)				−0.10 (0.14)		
Non Immigrant & SES Decreased		−0.39 (0.08) ***				−0.30 (0.11) *		
Immigrant & SES Stable		−0.30 (0.08) ***				0.43 (0.07) ***		
Immigrant & SES Increased		−0.32 (0.20)				0.56 (0.20) *		
Immigrant & SES Decreased		−0.66 (0.17) ***				0.18 (0.21)		
*(3) Interaction Between Immigrant & Poverty Direction (Reference group: Non Immigrant & Stable Poverty Status)*								
Non Immigrant & Improved Poverty Status			−0.48 (0.11) ***				−0.43 (0.10) ***	
Non Immigrant & Worsened Poverty Status			−0.56 (0.12) ***				−0.50 (0.12) **	
Immigrant & Stable Poverty Status			−0.34 (0.07) ***				0.36 (0.06) ***	
Immigrant & Improved Poverty Status			−0.54 (0.19) **				0.20 (0.15)	
Immigrant & Worsened Poverty Status			−0.55 (0.17) **				0.58 (0.17) ***	
*(4) Interaction Between Immigrant & Duration*								
Immigrant				−0.33 (0.07) ***				0.42 (0.07) ***
Times in Poverty				−0.20 (0.02) ***				−0.13 (0.02) ***
Immigrant * Times in Poverty				0.16 (0.03) ***				0.10 (0.03) ***
**Variance Components**								
Within-person	4.50 (0.02) ***	4.47 (0.02) ***	4.48 (0.02) ***	4.46 (0.02) ***	4.32 (0.02) ***	4.29 (0.02) ***	4.29 (0.02) ***	4.28 (0.02) ***
Level 2-between person								
In initial status	7.84 (0.05) ***	7.94 (0.06) ***	7.93 (0.06) ***	7.88 (0.05) ***	7.56 (0.05) ***	7.66 (0.05) ***	7.66 (0.05) ***	7.60 (0.05) ***
In rate of change	2.07 (0.03) ***	2.09 (0.03) ***	2.08 (0.03) ***	2.07 (0.03) ***	1.48 (0.03) ***	1.48 (0.03) ***	1.48 (0.03) ***	1.47 (0.03) ***
Covariance	−0.52 (0.01) ***	−0.50 (0.01) ***	−0.51 (0.01) ***	−0.51 (0.01) ***	−0.36 (0.01) ***	−0.34 (0.01) ***	−0.34 (0.01) ***	−0.35 (0.01) ***
Level 3-between school								
In initial status	3.45 (0.11) ***	3.59 (0.11) ***	3.59 (0.11) ***	3.38 (0.11) ***	3.22 (0.10) ***	3.37 (0.10) ***	3.38 (0.10) ***	3.14 (0.10) ***
In rate of change	1.08 (0.04) *	1.14 (0.04) ***	1.12 (0.04) ***	1.08 (0.04) *	1.00 (0.03)	1.02 (0.03)	1.01 (0.03)	0.99 (0.03)
Covariance	−0.64 (0.03) ***	−0.52 (0.03) ***	−0.53 (0.03) ***	−0.65 (0.03) ***	−0.61 (0.03) ***	−0.52 (0.03) ***	−0.54 (0.03) ***	−0.61 (0.03) ***
**Model Fit Statistics**								
Deviance (=−2 log-likelihood)	512,632.0	513,944.6	513,847.6	512,336.2	506,666.2	507,656.9	507,589.7	506,235.8
AIC	512,716.0	514,020.6	513,923.6	512,404.2	506,750.2	507,733.0	507,665.7	506,303.8
BIC	513,103.4	514,371.2	514,274.1	512,717.8	507,137.6	508,083.5	508,016.2	506,617.4
R-Squared	0.1942	0.1733	0.1784	0.1987	0.1977	0.1780	0.1817	0.2014

*Note.* Standard errors are in parentheses. Model also controls for child’s characteristics: being a boy, race/ethnicity (i.e., non-Hispanic Black, Hispanic, Asian and other racial/ethnic group), low birth weight, and attending center-based care before kindergarten; family characteristics: mother married at birth, having siblings present, number of family members under age 18 at home, living in single-parent family, parental educational expectations, parental participating in school events, home learning activities, and region and location of residence. AIC = Akaike’s information criterion; BIC = Bayesian information criterion. * *p* < 0.05, ** *p* < 0.01, *** *p* < 0.001.
